# Kinome profiling of peripheral blood mononuclear cells collected prior to vaccination reveals biomarkers and potential mechanisms of vaccine unresponsiveness in pigs

**DOI:** 10.1038/s41598-020-68039-6

**Published:** 2020-07-14

**Authors:** Sean W. L. Lipsit, James Wilkinson, Erin Scruten, Antonio Facciuolo, Connor Denomy, Philip J. Griebel, Anthony Kusalik, Graham Plastow, Scott Napper

**Affiliations:** 1 Vaccine and Infectious Disease Organization-International Vaccine Centre (VIDO-InterVac), University of Saskatchewan, Saskatoon, SK Canada; 2 Department of Biochemistry, Microbiology and Immunology, University of Saskatchewan, Saskatoon, SK Canada; 3 Department of Agricultural, Food and Nutritional Science, University of Alberta, Edmonton, AB Canada; 4 School of Public Health, University of Saskatchewan, Saskatoon, SK Canada; 5 Department of Computer Science, University of Saskatchewan, Saskatoon, SK Canada; 6 Illumina Centre, Great Abington, Cambridge, UK

**Keywords:** Predictive markers, Vaccines

## Abstract

Inter-individual variance in host immune responses following vaccination can result in failure to develop protective immunity leaving individuals at risk for infection in addition to compromising herd immunity. While developing more efficacious vaccines is one strategy to mitigate this problem, predicting vaccine responsiveness prior to vaccination could inform which individuals require adjunct disease management strategies. To identify biomarkers of vaccine responsiveness, a cohort of pigs (n = 120) were vaccinated and pigs representing the high (n = 6; 90th percentile) and low (n = 6; 10th percentile) responders based on vaccine-specific antibody responses following vaccination were further analyzed. Kinase-mediated phosphorylation events within peripheral blood mononuclear cells collected prior to vaccination identified 53 differentially phosphorylated peptides when comparing low responders with high responders. Functional enrichment analysis revealed pro-inflammatory cytokine signaling pathways as dysregulated, and this was further substantiated by detection of higher (p < 0.01) concentrations of interferon-gamma in plasma of low responders compared to high responders prior to vaccination. In addition, low responder pigs with high plasma interferon-gamma showed lower (p < 0.01) birth weights than high responder pigs. These associations between vaccine responsiveness, cytokine signaling within peripheral immune cells, and body weight in pigs provide both evidence and insight into potential biomarkers for identifying low responders to vaccination.

## Introduction

Infectious diseases currently represent the largest source of economic losses to the livestock industry^1^. With increased regulation against antibiotic usage in livestock, there is an even greater need to manage infectious diseases through alternative strategies like vaccination^2^. An inherent challenge with vaccination is the quantitative and qualitative variability in immune responses. Individuals that fail to generate an immune response to vaccination remain at risk for infection and compromise the protection afforded through herd immunity^3^. Unfortunately, the empirical approach that is typically taken for vaccine development does little to enhance our understanding of the mechanisms or molecular events promoting differential immune responses to vaccination. Biomarkers predictive of vaccine responsiveness could enable more strategic management of both vaccination programs and animal health. Furthermore, understanding the molecular events associated with vaccine responsiveness could guide the development of more effective vaccines.

Within human and livestock populations, biomarkers capable of predicting vaccine immunogenicity and efficacy could be used to reduce the costs associated with vaccine production and testing^4^. This need has spawned ‘systems vaccinology’ approaches that define the host factors contributing to immune responses to a variety of vaccines^5–9^. As vaccine-induced immune responses have shown significant heritability in animals^10^, identifying traits indicative of immune responsiveness could likely be applied for marker-assisted selection of animals^11^. In humans, a genome-wide association study attributed ~ 30% of the individual variability in the humoral immune response to measles vaccine to polymorphisms in immune response genes and human leukocyte antigen alleles^12^. Other genome-wide assessments of vaccine-induced immune responses in humans have identified associations with polymorphisms in antigen presentation proteins and lymphocyte receptors such as toll-like receptors^13^, signaling lymphocyte activation molecule^14^, cytokines, and cytokine receptors^15,16^.

While genetic variability can influence individual immune responsiveness to vaccination, the situation is further complicated by dynamic variables including nutritional, situational, environmental, and an individual’s health status. There are populations in which vaccines have proven to be less effective, specifically the elderly^17^, the young^18^, the obese^19,20^, and individuals with health factors that influence immune function, including stress^21^, autoimmunity^22,23^, and infection^24^. As such, an individual’s response to vaccination may be compromised regardless of their genetic predispositions. This necessitates the identification of more dynamic correlates of vaccine responsiveness. Dynamic variables affecting vaccine-induced immune responses have prompted immune-profiling efforts through transcriptional analysis, which have begun to illustrate transcriptional signatures associated with responses to various vaccines. For example, transcriptomic analysis of whole blood combined with polychromatic flow cytometry has identified both gene expression markers and immune cell-types characterizing vaccine-induced immune response processes to yellow fever-17D vaccination^8,25^. Similarly, transcriptional analysis of peripheral blood mononuclear cells (PBMCs) has revealed differential gene expression as correlates of hemagglutination inhibition titer responses at early time-points following influenza vaccination^5^. While transcriptional and cellular events have been explored in the hours following vaccination of livestock animals^26^, biomarkers that are predictive of the vaccine-induced immune responses prior to vaccination remain elusive, and the mechanisms that underlie vaccine responsiveness have yet to be fully defined.

Characterizing global cellular kinase activity (kinome analysis) is emerging as a powerful approach for understanding complex biology. Dynamic kinases catalyze phosphorylation events that regulate numerous cellular processes and often initiate a rapid functional change within cells. Species-specific peptide arrays used for high throughput kinome characterizations have delineated the complex biology associated with immune competence^27^ and biomarker discovery^28^. Within economically important livestock species, kinome analysis has revealed differential phosphorylation of host signaling pathways in response to mycotoxin consumption in pigs^29^ and *Mycobacterium avium* subsp. *paratuberculosis* infection in cattle^30,31^. As such, the genetic and dynamic traits influencing vaccine responsiveness may be better understood by characterizing kinase activity within immune cells at the time of vaccination.

Previously, transcriptional analysis was conducted on PBMCs collected from the pigs used in this study prior to vaccination^32^. Comparative analysis between the high and low responders prior to vaccination could only detect statistically significant differences in gene expression after vaccination. Given the ability of kinome analysis to investigate mechanisms of host phenotypes, kinome analysis was used to analyze cell signaling events within PBMCs collected from pigs immediately prior to vaccination. Pigs were subsequently stratified on the basis of serum *Mycoplasma hyopneumoniae* (*M. hyopneumoniae*)-specific IgG titer induced by RespiSure-One vaccination and grouped as high (HR) and low (LR) responders. While specific T cell responses can be used as a metric for vaccine responses, the current study uses serum IgG titers as the metric for vaccine responsiveness. Kinome analysis of PBMC lysates revealed different phosphorylation patterns when comparing cohorts of HR and LR pigs, where phosphorylation differences implicated pro-inflammatory cytokine signaling. Subsequent analysis of plasma cytokine concentrations confirmed higher concentrations of plasma interferon-gamma (IFNγ) in LR pigs compared to HR pigs at the time of vaccination. Information obtained from kinome analysis was then used to improve the understanding of host factors influencing vaccine responsiveness and to search for a specific biomarker that was predictive of vaccine responsiveness at the time a vaccine is administered.

## Results

### Differential antibody responses to vaccination

**Figure 1:**
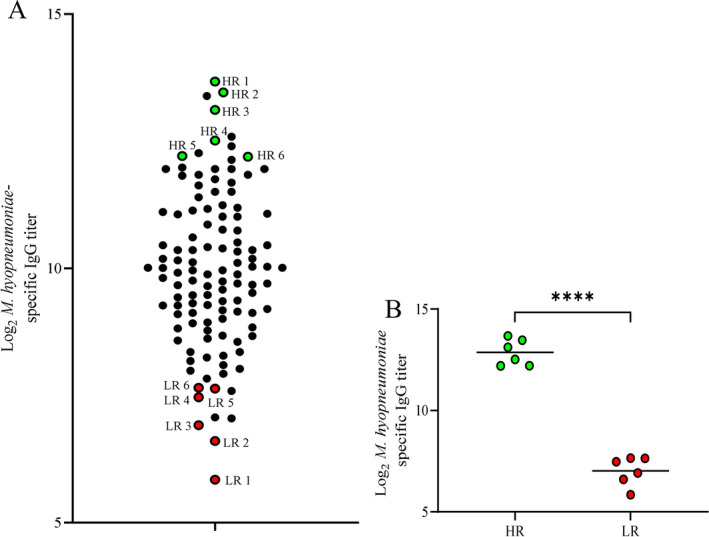
Variability in vaccine responsiveness in a population of pigs vaccinated with RespiSure-One and selecting high and low responders. (**A**) Serum *M. hyopneumoniae*-specific IgG titer in vaccinated pigs. High responders (HR; green) and low responders (LR; red) were selected from the cohort. (**B**) Serum *M. hyopneumoniae*-specific IgG titer of HR and LR pigs 35 days following primary vaccination; lines represent the mean. n = 117 in (**A**) and n = 6 for both HR and LR groups in (**B**); ****p < 0.0001, unpaired two-tailed Student’s t-test.

Pigs were vaccinated with an *M. hyopneumoniae* vaccine (Respisure-One) and serum IgG antibody-responses were quantified by IDEXX ELISA at 11 days after booster vaccination. There was a broad variation in serum *M. hyopneumoniae*-specific IgG titer responses to the vaccine, with log_2_ titer values ranging from 5.85 to 13.67. Based on titers and sample availability, six pigs above the 90th percentile and six pigs below the 10th percentile of serum *M. hyopneumoniae*-specific IgG titer were selected as high (HR) and low (LR) responders, respectively (Fig. 1a). The HR and LR pigs differed (p < 0.0001) in serum *M. hyopneumoniae*-specific IgG titer (Fig. 1b) and were selected for subsequent analyses to determine biomarkers of vaccine responsiveness.

### Kinome analysis of high and low responders

**Figure 2:**
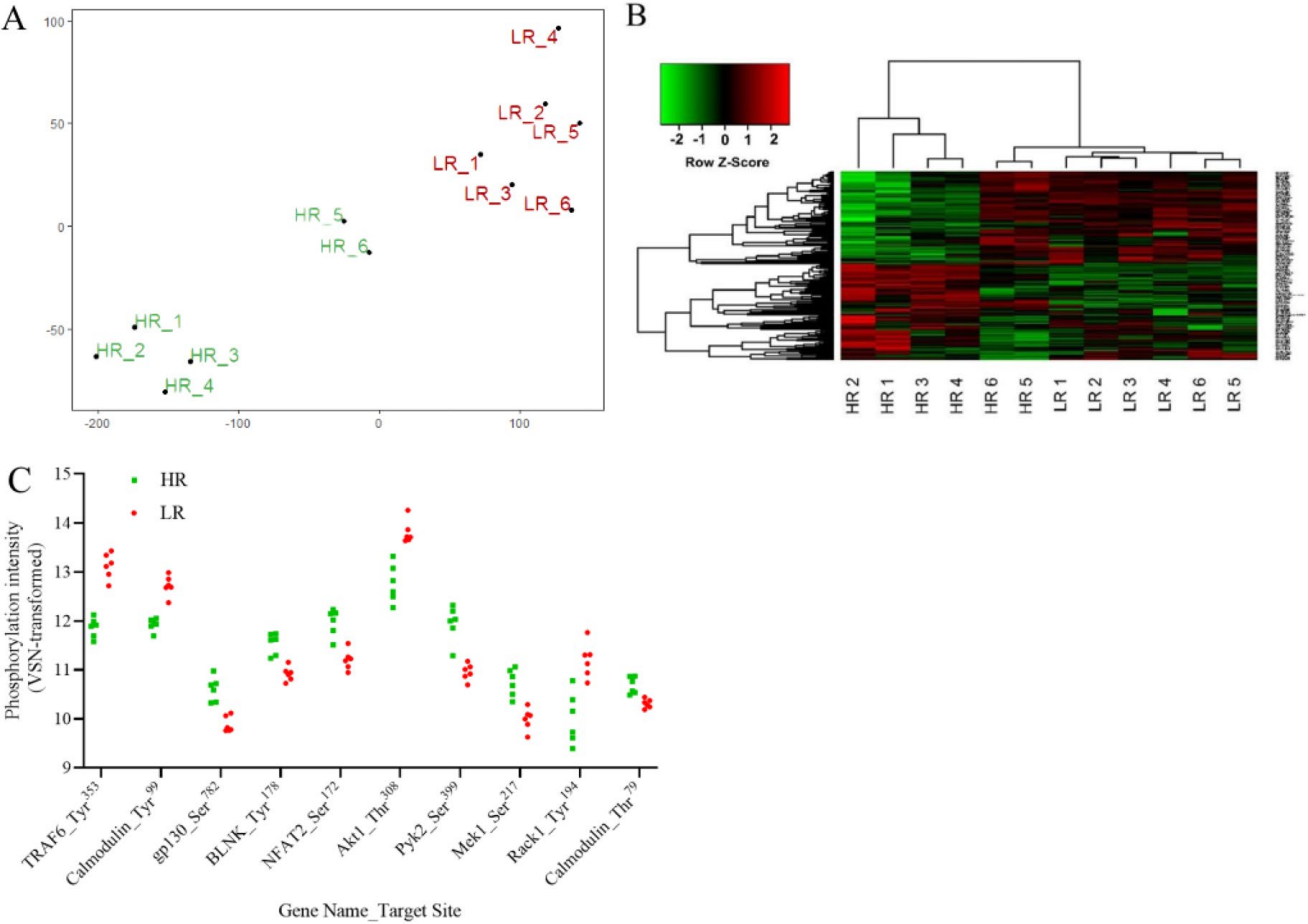
Differential phosphorylation patterns within PBMCs collected from high and low responder pigs prior to vaccination. (**A**) t-Distributed Stochastic Neighbor Embedding analysis and (**B**) Hierarchical clustering of high (HR) and low (LR) responders based on differential phosphorylation patterns in PBMCs collected prior to vaccination. (**C**) Top 10 most differentially phosphorylated peptides between HR (green) and LR (red) pigs prior to vaccination. n = 6 for both HR and LR groups.

Kinome analysis was performed on PBMCs isolated immediately prior to primary vaccination and restricted to animals in the HR and LR cohorts. Of the 282 unique peptide targets represented on the arrays, 161 were consistently (χ^2^ > 0.01) phosphorylated among the nine technical replicates. Therefore, characterization of the HR and LR kinome profiles was restricted to these 161 consistently phosphorylated peptides. t-distributed Stochastic Neighbour Embedding (t-SNE) analysis of HR and LR datasets was used to cluster similar kinome profiles of pigs. t-SNE of the kinome data grouped HR and LR pigs based on their serum IgG titer following booster vaccination (Fig. 2a). Hierarchical clustering based on differential phosphorylation of peptides prior to primary vaccination showed a clear clustering of pigs reflecting their vaccine responsiveness 11 days after booster vaccination (Fig. 2b). In both t-SNE and hierarchical clustering analyses, HR 5 and HR 6 show distinct clustering separate from HR 1–4 and LR 1–6 which may suggest unique signaling events within these PBMCs prior to vaccination.

Consistent with the observation that HR and LR pigs have unique phosphorylation patterns prior to vaccination, 53 individual peptides were found to be differentially phosphorylated (p < 0.05) when comparing HR and LR pigs (Table 1). Of the peptides represented on the array, the peptides which showed the greatest difference on the basis of statistical value included innate immunity-related genes and cell signaling mediators (Fig. 2c). Individual peptides differentially phosphorylated when comparing HR and LR pigs were used for pathway analysis to identify cell signaling pathways in which the peptides may be involved.

**Table 1:** Differentially phosphorylated peptides of PBMCs collected prior to vaccination between HR and LR pigs.

Name	Target site	UniProt ID	FC	p-value
TRAF6	Tyr^353^	Q9Y4K3	− 2.39	4.53E − 06
Calmodulin	Tyr^99^	P62158	− 1.73	3.44E − 05
gp130	Ser^782^	P40189	1.65	2.41E − 04
BLNK	Tyr^178^	Q8WV28	1.54	2.92E − 04
NFAT2	Ser^172^	O95644	1.71	3.26E − 04
Akt1	Thr^308^	P31749	− 2.06	4.26E − 04
Pyk2	Ser^399^	Q14289	1.99	4.72E − 04
Mek1	Ser^217^	Q02750	1.68	5.27E − 04
Rack1	Tyr^194^	P63244	− 2.28	1.38E − 03
Calmodulin	Thr^79^	P62158	1.29	1.89E − 03
MAVS	Ser^233/234^	Q7Z434	− 1.26	1.93E − 03
PPP2Cα	Thr^307^	P67775	− 1.72	1.99E − 03
Grb10	Ser^150^	Q13322	1.58	2.43E − 03
Akt1	Thr^450^	P31749	1.37	2.71E − 03
Syk	Tyr^348^	P43405	1.40	4.15E − 03
Kit	Tyr^936^	P10721	1.49	4.61E − 03
Caspase-8	Tyr^448^	Q14790	1.39	5.16E − 03
PKACα	Thr^197^	P17612	− 2.10	5.43E − 03
4E-BP1	Thr^46^	Q13541	1.31	5.95E − 03
TNIK	Thr^181^	Q9UKE5	2.10	8.59E − 03
p70S6K	Ser^447^	P23443	− 1.56	8.69E − 03
Kit	Tyr^568/570^	P10721	1.52	8.85E − 03
IKK-α	Ser^473^	O15111	− 1.46	9.16E − 03
p53	Ser^15^	P04637	− 1.36	9.61E − 03
Smad6	Ser^435^	O43541	− 2.64	1.18E − 02
PKCε	Thr^566^	Q02156	− 1.24	1.33E − 02
Kit	Tyr^721^	P10721	1.26	1.40E − 02
IRAK1	Thr^100^	P51617	− 1.44	1.42E − 02
NFAT2	Ser^245^	O95644	− 1.56	1.60E − 02
Cofilin 1	Ser^2^	P23528	1.51	1.66E − 02
HSP60	Ser^70^	P10809	− 1.35	1.67E − 02
PDGFRβ	Tyr^740^	P09619	1.48	1.80E − 02
Smad3	Thr^179^	P84022	1.30	1.81E − 02
Lyn	Tyr^396^	P07948	1.47	1.82E − 02
TBK1	Ser^172^	Q9UHD2	− 1.40	1.85E − 02
JAK1	Tyr^220^	P23458	− 2.91	1.95E − 02
STAT6	Tyr^641^	P42226	− 1.78	1.97E − 02
HSP27	Ser^78^	P04792	1.92	2.07E − 02
p300	Ser^2279^	Q09472	− 1.27	2.12E − 02
Cdc42	Tyr^32^	P60953	− 1.48	2.28E − 02
IKK-β	Tyr^188^	O14920	1.30	2.63E − 02
Smad1	Ser^214^	Q15797	1.41	2.96E − 02
MSK2	Ser^360^	O75676	− 1.43	3.24E − 02
Bcl-2	Ser^87^	P10415	− 1.27	3.66E − 02
K8	Tyr^267^	P05787	− 2.01	3.68E − 02
CDK2	Thr^160^	P24941	− 1.38	3.83E − 02
fyn	Tyr^531^	P06241	1.33	4.03E − 02
CREB	Ser^117^	P16220	1.27	4.11E − 02
IKK-β	Tyr^199^	O14920	− 1.30	4.52E − 02
ERK3	Ser^189^	Q16659	− 1.86	4.66E − 02
IKK-γ	Ser^43^	Q9Y6K9	− 1.33	4.73E − 02
P27kip1	Tyr^74^	P46527	− 1.55	4.90E − 02
Sek1	Ser^80^	P45985	− 1.28	4.94E − 02

Fold-change (FC) in HR relative to LR.

### Pathway over-representation analysis

To further analyze the variations in signaling within the PBMCs of HR and LR pigs collected prior to vaccination, the 53 differentially phosphorylated peptides were subjected to over-representation analysis (ORA) using InnateDB. Of the 53 peptides queried, InnateDB designated 27 (51%) to belong to *Innate Immune System* (Table 2). Within the pathway analysis dataset, there was a high representation of cytokine signaling entities, such as *TNFalpha, IL-7 signaling, IL-2, IL-3, and IL-6,* in addition to innate immunity signaling pathways including *RANKL* signaling and *JAK-STAT pathway and regulation* (Table 2). Altogether, ORA of the differentially phosphorylated genes between HR and LR suggests differential signaling of pro-inflammatory cytokines and innate immune signaling in the PBMCs of HR and LR pigs prior to vaccination.

**Table 2:** Pathway overrepresentation analysis of differentially phosphorylated peptides in PBMCs collected from HR and LR pigs prior to vaccination.

Pathway name	Source name	Pathway p-value (corrected)	Number of uploaded genes for this entity	Number of genes in InnateDB for this entity
Innate Immune System	REACTOME	4.11E − 20	27	563
Signaling by Interleukins	REACTOME	8.77E − 17	15	110
RANKL	NETPATH	1.22E − 16	14	84
Immune System	REACTOME	2.42E − 16	30	1,127
BCR	NETPATH	3.45E − 16	16	157
Fc epsilon receptor (FCERI) signaling	REACTOME	1.40E − 15	16	173
JAK-STAT pathway and regulation	INOH	2.04E − 15	18	273
TNFalpha	NETPATH	2.05E − 15	18	270
VEGF signaling pathway	INOH	2.08E − 15	16	183
EPO signaling pathway	INOH	2.18E − 15	16	181
IL-7 signaling	INOH	2.28E − 15	16	180
Leptin	NETPATH	7.64E − 15	13	96
IL2	NETPATH	3.85E − 14	12	81
IL3	NETPATH	5.66E − 14	12	84
IL6	NETPATH	6.13E − 14	12	85
Prostate cancer	KEGG	1.18E − 13	12	90
EGFR1	NETPATH	1.33E − 12	19	472
BCR signaling pathway	PID NCI	4.33E − 12	10	63
MyD88-independent cascade	REACTOME	8.80E − 12	11	97
TRIF-mediated TLR3/TLR4 signaling	REACTOME	8.80E − 12	11	97
Toll Like Receptor 3 (TLR3) Cascade	REACTOME	8.80E − 12	11	97
Osteoclast differentiation	KEGG	9.25E − 12	12	133
Pathways in cancer	KEGG	9.59E − 12	16	329
Cytokine Signaling in Immune system	REACTOME	9.60E − 12	15	267
Signaling by NGF	REACTOME	9.86E − 12	15	271

The top 25 pathways are presented.

### Cytokine profiling of high and low responders

**Figure 3:**
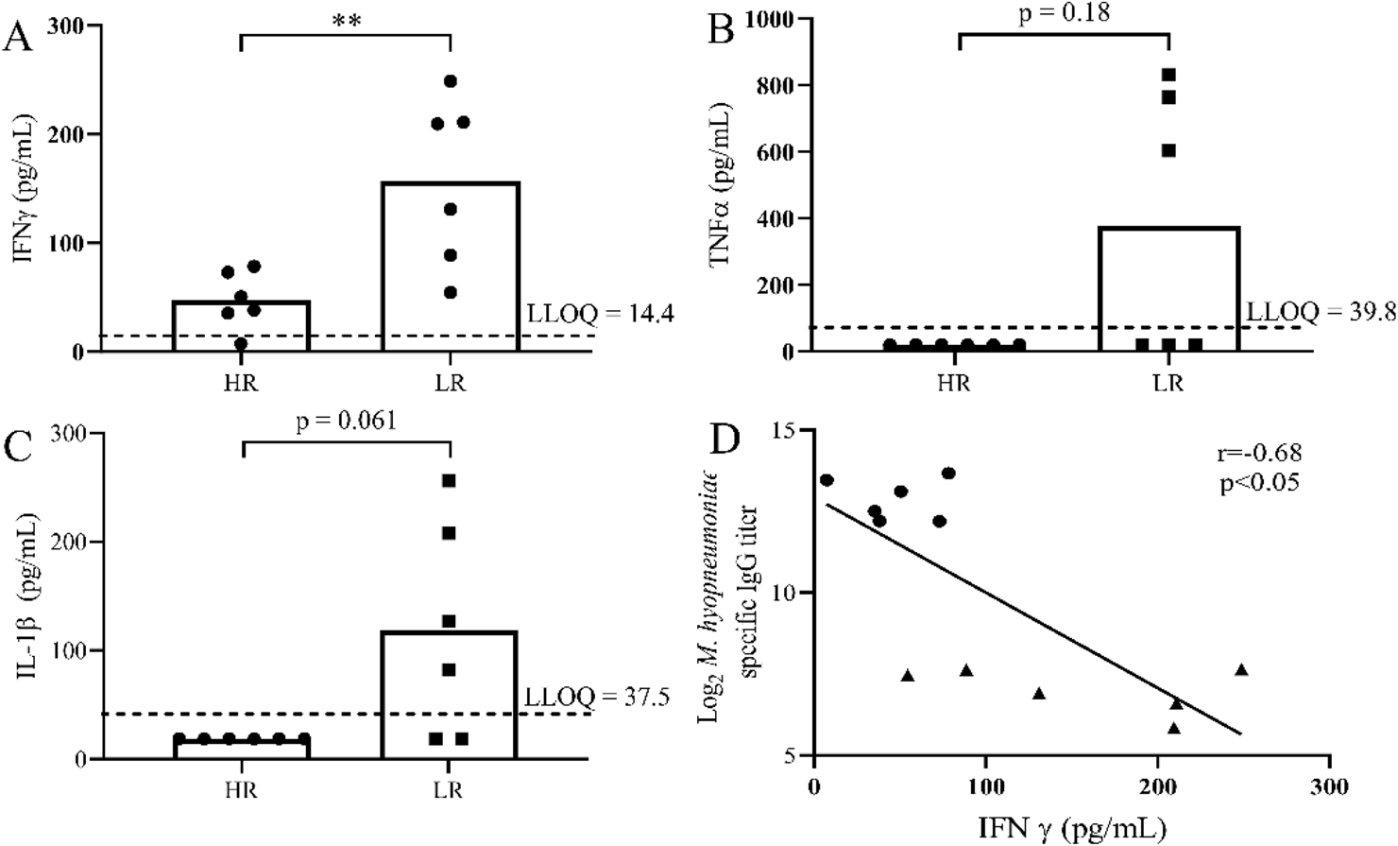
Elevated pro-inflammatory cytokines in low responder plasma compared to high responders prior to vaccination. Concentrations of (**A**) IFNγ, (**B**) TNFα, and (**C**) IL-1β within the plasma of high (HR) and low (LR) responder pigs collected prior to vaccination. (**D**) Correlation analysis of plasma IFNγ concentrations prior to vaccination and post-vaccination serum *M. hyopneumoniae*-specific IgG titer for HR (circles) and LR (triangles). Bars represent the mean. n = 6 for both HR and LR groups. Samples below the limit of lower quantification (LLOQ) were included as ½ the LLOQ for each assay. **p < 0.01, Mann–Whitney U-test.

To further explore the observation that pro-inflammatory cytokine signaling of HR and LR pigs was differentially regulated, nine cytokines (IFNα, IFNγ, IL-1β, IL-6, IL-8, IL-12, IL-13, IL-17α and TNFα) were assayed in plasma collected prior to primary vaccination of HR and LR pigs. LR pigs had higher (p = 0.0087) plasma concentration of IFNγ compared to HR pigs (Fig. 3a). A possible difference in TNFα (p = 0.18) (Fig. 3b) and IL-1β (p = 0.061) (Fig. 3c) plasma concentrations was also observed when comparing LR and HR pigs. HR and LR pigs showed no significant difference (p > 0.05) in IFNα, IL-6, IL-8, IL-12, IL-13, or IL-17α plasma concentrations prior to primary vaccination. Finally, within the HR and LR pigs, plasma IFNγ concentration prior to primary vaccination was negatively correlated (r = − 0.68, p < 0.05) with serum *M. hyopneumoniae*-specific IgG titer (Fig. 3d). Correlation analyses were not conducted for TNFα and IL-1β due to the low number of data-points available for these cytokines in the HR and LR cohorts.

### Birth weight differences between high and low responders

Bodyweight of pigs, specifically birth weight, has been used as an indicator of growth and survival^33^ and has been previously associated with vaccine responsiveness^34^. The design of this study deliberately excluded the naturally-occurring extremes of piglet birth weight by including only the six piglets per litter with average-litter birth weight. This reduction in birth weight variation may have limited the analysis of birth weight as a biomarker for predicting vaccine responsiveness.

**Figure 4:**
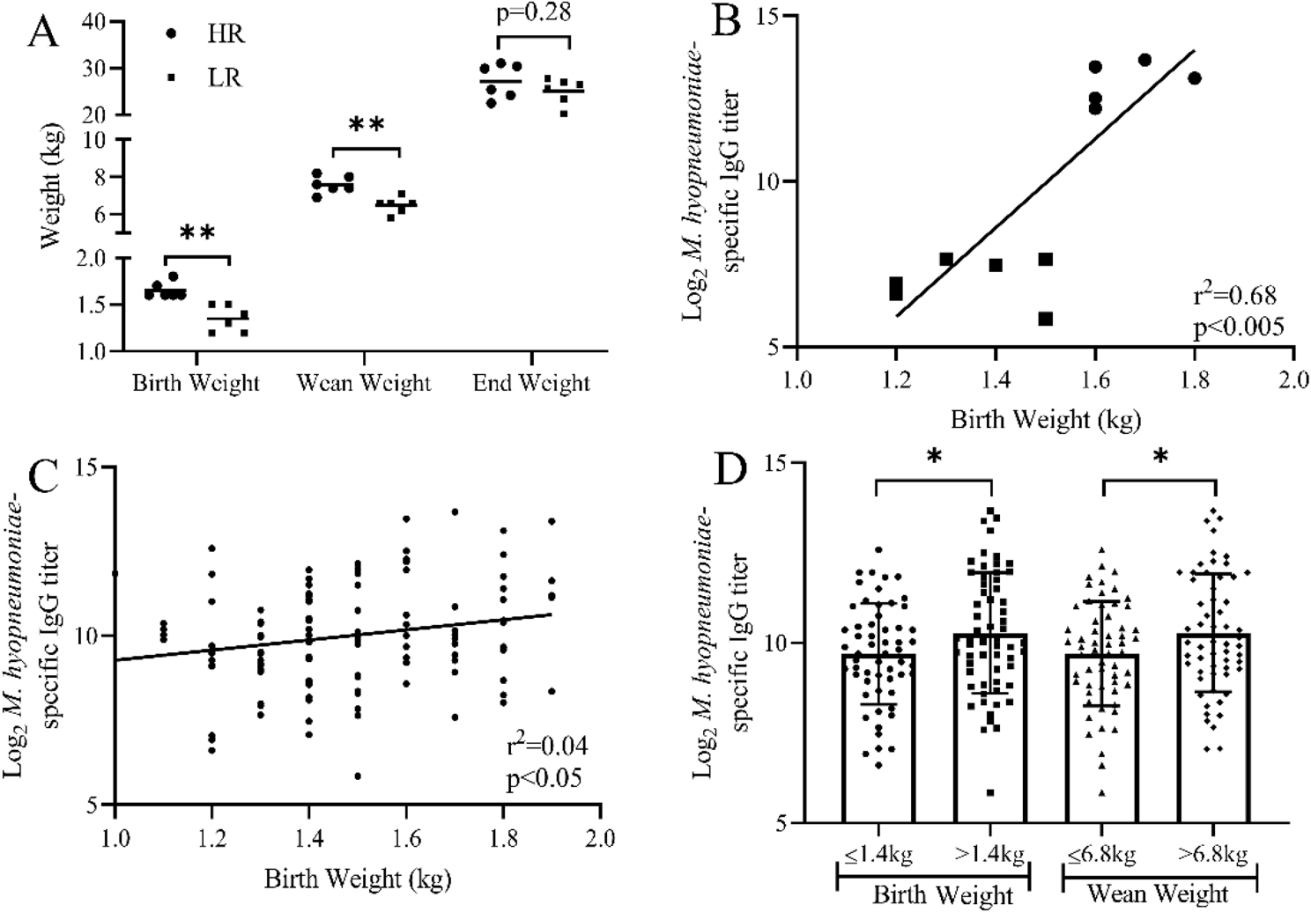
Vaccine-induced antibody responses correlate with both birth and weaning bodyweights of high and low responder pigs. (**A**) Bodyweight of high (HR; circles) and low (LR; squares) responder pigs at birth, weaning, and at the end of experiment. (**B**) Correlation analysis of birth weight and serum *M. hyopneumoniae*-specific IgG titer for HR (circles) and LR (squares) pigs. (**C**) Correlation analysis of birth weight and serum *M. hyopneumoniae*-specific IgG titer for the sample cohort. (**D**) Serum *M. hyopneumoniae*-specific IgG titer of pigs with median birth weight less than (circles) or greater than (squares) 1.4 kg, and pigs withs median wean weight less than (triangles) or greater than (diamonds) 6.8 kg. Data represents mean in (**A**) and mean ± SD in (**D**). Line represents best-fit line of linear regression in (**B**) and (**D**). n = 6 for both HR and LR groups in (**A**) and (**B**), n = 117 in (**C**), and n ≥ 57 for each group in (**D**). *p < 0.05, **p < 0.01, unpaired two-tailed Student’s t-test.

Within the sample population, the range of birth weights, weaning weights, and end weights of the pigs was 1.0–1.9 kg, 4.6–10.0 kg, and 19.0–37.7 kg, respectively. LR pigs had lower birth (p = 0.001) and weaning (p = 0.0018) weights compared to HR pigs (Fig. 4a). At the end of the experiment (Day 63), a difference (p = 0.28) in weight between HR and LR pigs was not observed, which may suggest that LR pigs did not have inherent long-term growth impairments (Fig. 4a). Within the HR and LR cohorts, there was a positive correlation (r^2^ = 0.68, p < 0.005) between birth weight and serum *M. hyopneumoniae*-specific IgG titer (Fig. 4b). While a correlation between birth weight and serum *M. hyopneumoniae*-specific IgG titer was not evident within the entire sample population (r^2^ = 0.04, p < 0.05) (Fig. 4c), pigs below the median birth weight (1.0–1.4 kg) had a lower (p = 0.046) serum *M. hyopneumoniae*-specific IgG titer than pigs above the median birth weight (1.5–1.9 kg) (Fig. 4d). Similarly, pigs with a weaning weight below the median weight (4.6–6.8 kg) had a lower (p = 0.0478) serum *M. hyopneumoniae*-specific IgG titer compared to pigs above the median wean weight (6.9–10 kg) (Fig. 4d).

### Correlation analysis of birth weight, plasma IFNγ, and vaccine responsiveness

**Figure 5:**
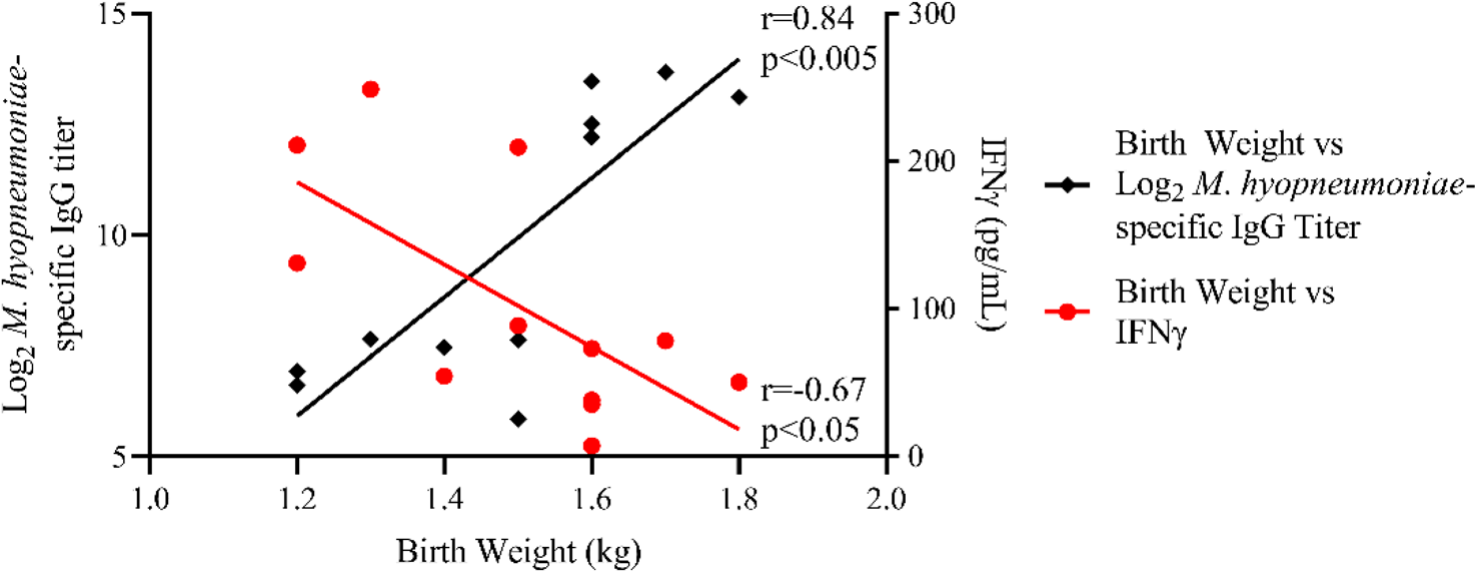
Birth weight of high and low responder pigs correlate with both vaccine responsiveness and plasma IFNγ prior to vaccination. Correlation analysis of birth weight and both serum *M. hyopneumoniae*-specific IgG titer (black) and plasma IFNγ concentrations (red) prior to vaccination in high (HR) and (LR) responders. n = 6 for both HR and LR groups. A single data-point represents a single animal for each analysis. Samples below the limit of lower quantification (LLOQ) were plotted as ½ the LLOQ.

Following observation of the positive correlation between birth weight and serum *M. hyopneumoniae*-specific IgG titer, and the negative correlation between plasma IFNγ concentrations and serum *M. hyopneumoniae*-specific IgG titer, correlation analysis of HR and LR birth weight and plasma IFNγ concentrations were found to be negatively correlated (r = − 0.67, p < 0.05) (Fig. 5). Collectively, pigs exhibiting high and low serum *M. hyopneumoniae*-specific IgG titer following vaccination show disparity in birth weight and plasma IFNγ concentrations prior to vaccination. This data provides evidence that the magnitude of vaccine-induced antibody responses may be predicted by biological parameters measured prior to or at the time of vaccination.

## Discussion

While vaccination is one of the most effective tools for preventing and managing infectious diseases, individuals who fail to develop an immune response following vaccination can remain susceptible to infection. The low responders have the potential to transmit infection to others in the population and reduce the proportion of protected individuals needed for herd immunity. Kinome analysis of PBMCs collected prior to primary vaccination led to the discovery of differentially phosphorylated peptides when comparing HR and LR pigs, demonstrating that vaccine responsiveness is associated with unique host cell signaling prior to vaccination. The kinome analysis results implicated differential pro-inflammatory cytokine signaling pathways as a difference between HR and LR pigs. This finding was further validated by analyzing the plasma cytokine concentrations in HR and LR pigs prior to primary vaccination. The analysis revealed differences in circulating plasma cytokine levels that significantly correlated with both animal birth weight and serum specific-IgG titer following booster vaccination. Biomarkers capable of predicting high and low responders prior to vaccination, such as body weight or plasma IFNγ concentrations, may facilitate the identification of low responders and eliminate the need to rely on post-vaccination analysis of antibody titers. The positive correlation between birth weight and vaccine-induced antibody titer within high and low responders may present a simple, immediate biomarker for potentially identifying low-responders at birth, where animals who exhibit low birth weight could potentially be at risk of having a reduced chance of developing a vaccine-induced immune response. Correlations between vaccine responsiveness and both piglet birth weight and plasma cytokine concentrations of high and low responders reveal differences that exist prior to vaccination that may have had long-term impacts on vaccine responses, even after two vaccinations.

Several studies have searched for biomarkers and investigated possible mechanisms of vaccine responsiveness by analyzing genetic^12,16^ and dynamic factors such as age^35,36^, weight^20^, and health status^21,24^. Other studies have provided insight into the molecular mechanisms influencing immune responses to vaccination through a variety of -omic approaches^5,7,8,26,32,37^. The current investigation identified biological differences in kinase-mediated signaling that were present in PBMCs collected prior to vaccination that discriminated high and low responders. Reduction analysis of the peptide phosphorylation patterns within the PBMCs clustered pigs on the basis of serum specific-IgG titers 11 days following booster vaccination which may suggest the cohorts had a predisposition towards a specific vaccine-induced antibody response. In contrast, transcriptional analysis of PBMCs collected from the same HR and LR pigs used in the current study was only able to discriminate these cohorts at two and six days following primary vaccination^32^. This may reflect the greater sensitivity of kinome analysis to identify cell signaling events in PBMCs at a post-transcriptional level that can directly influence vaccine responsiveness.

In addition to identifying to potential biomarkers of vaccine responsiveness, the kinome analysis generates hypotheses regarding the biological processes that may influence vaccine responses. Within the PBMCs collected prior to vaccination, differences in phosphorylation events between HR and LR pigs revealed altered pro-inflammatory signaling, a finding consistent with the higher (p = 0.0087) plasma IFNγ concentration in LR pigs compared to HR pigs. This finding is in agreement with other evidence that pro-inflammatory events prior to vaccination are negatively associated with vaccine responsiveness. In an African population of human subjects vaccinated with the yellow fever 17D vaccine, the cohort with low YF-17D specific-neutralizing antibodies following vaccination displayed greater frequencies of pro-inflammatory monocytes and exhausted natural killer cells prior to vaccination^38^. Fourati et al*.* reported that seroconversion following hepatitis B virus vaccination of humans was negatively correlated with baseline pro-inflammatory signaling pathways, activated innate immune cells, and upregulation of inflammatory cytokines^39^. The pro-inflammatory cytokine TNFα has also been proposed as a biomarker of vaccine responsiveness in both humans and mice as increased serum TNFα negatively correlated with serum antibody responses and in vitro B-cell responses to stimulation with CpG oligodeoxynucleotides^40,41^. Consistent with these results, LR pigs showed a tendency towards greater levels of TNFα (p = 0.18) and IL-1β (p = 0.061) compared to HR pigs. The detection of elevated IFNγ, TNFα, and IL-1β in plasma of LR pigs supports the conclusion that circulating pro-inflammatory cytokines prior to vaccination are associated with reduced vaccine-induced antibody responses in pigs to RespiSure-One. However, the current study uses total serum IgG titers following two vaccinations as the only measure of vaccine responsiveness. Additional studies are required to determine if antibody isotypes were also qualitatively different and if T-cell responses were similarly reduced following vaccination.

Further studies are required to determine if IFNγ has a direct impact on vaccine responsiveness. IFNγ is involved in the upregulation of major histocompatibility complex class I and II, activation of macrophages, and production of pro-inflammatory cytokines. IFNγ is a crucial cytokine required for differentiating naïve CD4 T cells into Th1 effector cells to mediate cellular immunity in response to antigens^42^. However, excessive concentrations of IFNγ have been found in humans and mice with various autoimmune diseases^43–45^, suggesting IFNγ concentrations must be closely controlled for optimal immune responses^42^. As hyper-responsive innate immune systems have been proposed to negatively affect adaptive immune-responses^46^, disproportionate production of pro-inflammatory cytokines may be detrimental to the host and it should be investigated whether elevated plasma IFNγ in low responders persists throughout the post-vaccination period. Pro-inflammatory responses have been linked to dysbiosis of the gut microbiome due to antibiotic perturbation, in which broad-spectrum antibiotic use was found to negatively affect H1N1-specific IgG1 and IgA responses following trivalent inactivated influenza vaccination in humans^47^. The use of anti-inflammatory drugs, such as metformin, has improved influenza vaccine-specific antibody responses^48^, and it is suspected that other molecules that reduce systemic inflammation may improve vaccine-induced antibody responses in humans^49^. Collectively, there is strong evidence that pro-inflammatory cytokines can impact cellular responses prior to vaccination which subsequently has a negative effect on vaccine responsiveness.

Low birth weight has been a biomarker for predicting overall health, as within litters of pigs, low birth weight is associated with a greater risk of the piglets being stillbirths^50^ and greater pre-weaning mortality^51^. This investigation identified an association between body weight and vaccine responsiveness, where LR pigs had lower (p < 0.005) weights at birth and weaning than HR pigs. This association was identified in spite of an experimental design that minimized variance in body weight by selecting pigs of average-litter birth weight. Therefore, the current results may underestimate the strength of the correlation between body weight at both birth and weaning with vaccine responsiveness. A correlation between serum *M. hyopneumoniae*-specific IgG titer and birth weight was not apparent within the entire study cohort, yet the correlation exists when focusing on the extremes of vaccine responsiveness. A positive relationship between antibody responses and birth weight is consistent with a growing body of evidence which suggests early immune development is crucial in determining the capacity of individuals to generate serum antibody responses to vaccines. Independent studies of humans vaccinated with hepatitis B virus or typhoid fever vaccines have associated low birth weight and reduced serum antibody levels for both vaccines^18,52^. After identifying a correlation between reduced anti-typhoid IgG following typhoid vaccination and low birth weight in a subpopulation of adolescents^53^, elevated pro-inflammatory marker, C-reactive protein, was observed in those individuals with low anti-typhoid IgG responses^54^. The current study also reveals an association between lower birth weights, elevated concentrations of pro-inflammatory cytokines in plasma, and decreased vaccine-induced antibody responses.

In the context of livestock herds, a biomarker, such as plasma cytokine concentrations, could aid in rapidly identifying low responders prior to vaccination. Phenotypic indicators such as birth weight could also be used to designate animals who are potentially at greater risk for infection. Tests capable of predicting vaccine responsiveness have the potential to influence herd management decisions and possibly provide biomarkers to improve animal breeding programs. While these findings on piglet birth weight and plasma cytokine expression need to be replicated in an independent herd and trial, the current study provides evidence that two concurrent parameters are associated with vaccine responsiveness in young pigs.

## Materials and methods

### Animal care and vaccination

The experimental protocol was approved by the University of Alberta Animal Care and Use Committee-Livestock (AUP00001125) and sample collection was in accordance with the Canadian Council on Animal Care guidelines. The animals used for this study have been described previously^32^. Twenty sows bore litters in which six pigs (three males and three females) with average-litter birth weight were selected. Pigs born from the sows that were not of average-litter birth weight were not recorded in this study, ultimately resulting in a cohort of 117 healthy *M. hyopneumoniae*-free pigs [(Large White × Landrace) × Duroc; 59 male, 58 female]. Three pigs died before the end of the study and were excluded from analyses. Pigs were weaned and weighed at 21 ± 2 days of age and subsequently grouped with littermates in nursery pens (n = 29–30 pigs/pen). No difference (Ordinary one-way ANOVA, p > 0.9999) in *M. hyopneumoniae*-specific IgG titer was found between pens. Pigs were monitored for changes in weight gain, behaviour, and physical injury throughout the experiment. Pigs were vaccinated intramuscularly with one dose (1 mL) of RespiSure-One (Zoetis, USA) at 28 days of age and given a booster vaccine at 52 days of age. The trial was terminated when pigs were 63 days of age. In order to address if *M. hyopneumoniae* was circulating in the herd, a nasal swab from each animal was tested on D0 prior to vaccination to which all animals tested negative for the bacterium. While the maternal serological status was not measured for this study, it was confirmed that at the time of this study and for several years prior to this study, animals within the facility had consistently tested as being *M. hyopneumoniae*-free. Additionally, the sows were not vaccinated with RespiSure-One to eliminate the concern that vaccine-induced maternal antibodies were transferred to the pigs used in this study.

### Serum and plasma collection, and PBMC isolation

Whole blood was collected from the jugular vein at 28 days of age (prior to primary vaccination) using 0.4% EDTA (Sigma-Aldrich) in Ca^2+^ and Mg^2+^-free phosphate-buffered saline (PBS). Whole blood was centrifuged for 20 min at 1,400 × g at 20 °C without brake. The buffy coat layer at the interface of the red blood cells and plasma was collected, diluted 1:2 with room temperature PBS + 0.1% EDTA, and layered onto 15 mL of isotonic Ficoll (GE Healthcare) and centrifuged for 20 min at 2,000 × g at 20 °C without brake. PBMCs at the interface of the plasma and isotonic Ficoll were collected and washed twice with ice-cold PBS + 0.1% EDTA and pelleted by centrifuging for 8 min at 300 × g, 4 °C with brake. Cells were washed a third time in PBS and pelleted by centrifugation for 8 min at 150 × g, 4 °C with brake. Viable cell counts were determined by trypan blue (Gibco) exclusion using a hemocytometer. PBMC pellets containing 10 × 10^6^ cells were flash-frozen in liquid nitrogen and stored at − 80 °C.

Whole blood was collected from the jugular vein into anticoagulant-free and EDTA-coated Vacutainer tubes (Becton Dickinson) for the collection of serum and plasma, respectively, at 28 days of age (prior to primary vaccination) and at 63 days of age (11 days after booster vaccination). Serum collection tubes were incubated for 30 min at room temperature. Serum and plasma tubes were centrifuged for 10 min at 15,000 × g, 4 °C. Samples were aliquoted and stored at − 80 °C.

### IgG ELISAs

Methods for determining IgG titer have been described previously^32^. Briefly, 1 mL of serum from each pig at 63 days of age (11 days after booster vaccination) were shipped to Biovet (Saint-Hyacinthe, QC, Canada) where serum *M. hyopneumoniae*-specific IgG titers were quantified using an IDEXX ELISA kit (Idexx Laboratories, Inc.). All *M. hyopneumoniae*-specific IgG titers were transformed using a z-score log_2_ scale.

### Selection of high and low responders

Pigs were stratified by *M. hyopneumoniae*-specific IgG titer and the six pigs clustering for which there was sufficient sample to perform both kinome profiling and cytokine analysis, were selected to represent the high and low vaccine responders. Several animals were excluded from LR and HR cohorts based on the availability of limited archived samples. These pigs were, however, included in all population analyses for both the serum *M. hyopneumoniae*-specific IgG titer stratification and the correlation analysis of birth weight and *M. hyopneumoniae*-specific IgG titer.

### Kinome analysis

The design, construction and application of the peptide arrays were performed using previously described protocols^55^. The arrays were fabricated by a commercial provider (JPT Innovative Peptide Solutions) and designed to include peptides representing phosphorylation events associated with a wide variety of signaling pathways. Each array includes nine technical replicates of 282 unique peptides. All kinome experiments were performed on the same day to minimize potential inter-assay variance.

All products were purchased from Sigma-Aldrich unless otherwise stated. Briefly, 10 × 10^6^ PBMCs were lysed with ice-cold lysis buffer (20 mM Tris–HCl [pH 7.5], 150 mM NaCl, 1 mM EDTA, 1 mM EGTA, 1% Triton X-100, 2.5 mM sodium pyrophosphate, 1 mM Na_3_VO_4_, 1 mM NaF, 1 μg/mL leupeptin, 1 μg/mL aprotinin, 1 mM PMSF) and incubated for 10 min on ice. Lysates were centrifuged for 10 min at 14,000 × g, 4 °C. Supernatant was combined with 8:1 activation mix [50% glycerol, 50 μM ATP (New England Biolabs), 60 mM MgCl_2_, 0.05% Brij-35, 0.25 mg/mL bovine serum albumin] for 10 min on ice. Samples were incubated on the peptide array for 2 h at 37 °C. Arrays were washed with PBS + 1% Triton X-100 and submerged in ProQ Diamond phosphoprotein stain (Invitrogen) and incubated for 1 h with agitation. Arrays were destained with 20% acetonitrile + 50 mM sodium acetate, pH 4.0 for 10 min. Arrays were washed with distilled deionized water and centrifuged for 5 min at 800 × g to remove excess moisture. Phosphorylation intensity was collected using a GenePix Professional 4200A Microarray Scanner at 532–560 nm with a 580 nm filter. Images were captured using the GenePix Pro 6.0 software (MDS) to collect spot intensity^30^.

Peptide-spot intensities were transformed using a variance-stabilizing normalization (VSN) method through the online software, PIIKA (https://saphire.usask.ca/saphire/piika/). Peptides that showed variation in technical replicates via Chi-squared test (χ^2^ < 0.01) were removed from subsequent analysis. Consistent technical replicates were averaged together, and fold-change (FC) for each peptide was calculated as $FC={2}^{d}, d={average}_{ HR intensity}-{average }_{LR intensity}$. The t-distributed stochastic neighbour embedding (t-SNE) analysis and hierarchical clustering were conducted using peptides with consistent phosphorylation (χ^2^ > 0.01). The t-SNE analysis was conducted using the R package Rtsne (https://github.com/jkrijthe/Rtsne)^56^ and visualized using ggplot2 (https://ggplot2.tidyverse.org)^57^. The t-SNE analysis was performed 100 times and the result with the lowest value of the objective function was selected. The construction of the heatmap using PIIKA has been described previously^58^. Hierarchical clustering was conducted using the Pearson correlation distance and McQuitty linkage. Peptides were considered differentially phosphorylated under two given criteria: first, the peptide was consistently phosphorylated according to the Chi-squared test and second, the VSN-transformed phosphorylation intensity of an individual peptide was significantly different (two-tailed Welch’s t-test for Unequal Variances, p < 0.05) between cohorts.

### Pathway over-representation analysis

Peptides that were differentially phosphorylated were subjected to pathway over-representation analysis (ORA) using InnateDB^59^, an online software program for querying datasets of genes/proteins against multiple curated databases to determine biologically relevant pathways. ORA was completed using the hypergeometric algorithm with Benjamani–Hochberg correction method, and pathways were considered statistically significant with a false discovery rate (FDR) of p < 0.05.

### Porcine-specific multiplex analysis

Plasma samples had undergone two freeze–thaw cycles prior to multiplex analysis. All incubations were done at room temperature with agitation at 750 rpm. Plates were covered in foil to reduce light exposure. All products are from Sigma-Aldrich unless otherwise stated. Following each incubation, plates were washed with PBS pH 7.4 (Gibco) + 0.5% Tween 20 using a Bio-Plex PRO II wash station (30-s soak, 3 cycles). Standards were diluted 1:4 in New Zealand pig serum to account for serum inhibitory effects while samples were diluted 1:2 and 1:4 in diluent (PBS, 1% New Zealand pig serum, 0.05% sodium-azide). Porcine-specific antibodies for interferon α (IFNα), IFNγ, interleukin-1β (IL-1β), IL-6, IL-8, IL-12, IL-13, IL-17α and tumor necrosis factor α (TNFα) were conjugated to individual BioPlex Max-Plex C magnetic beads (BioRad) following the manufacturer’s instructions. TNFα multiplex analysis was conducted on a separate plate to avoid cross-reactivity.

Diluted porcine plasma was incubated for 1 h with 1,200 beads/well in a Fluortrac 200 96F microplate (Greiner Bio-One). Following one wash, samples were incubated for 30 min with biotinylated antibodies specific for the corresponding porcine-cytokine. Following another wash, samples were incubated for 30 min with 5 μg/mL streptavidin R-phycoerythrin conjugate (ThermoFisher Scientific). After a final wash, samples were incubated for 5 min with TE buffer (50 mM Tris, 25 mM EDTA, pH 8.0). Plates were read on a BioPlex 200 reader (Bio-Rad Laboratories Inc.) with the settings “50 beads per region, 45-s time-out, and 60 μL volume”. All replicates and dilution factors for each animal were averaged together for a final concentration. Technical replicates below the lower limit of quantification (LLOQ) were not calculated in the average result. Samples below the limit of detection were recorded as ½ the LLOQ value. LLOQ values for all cytokines are given: IFNα (1.57 pg/mL), IFNγ (14.4 pg/mL), IL-1β (37.5 pg/mL), IL-6 (40.3 pg/mL), IL-8 (2.72 pg/mL), IL-12 (39.1 pg/mL), IL-13 (40.0 pg/mL), IL-17α (15.5 pg/mL), and TNFα (39.8 pg/mL).

### Data and statistical analysis

All data analysis and data visualization were performed using GraphPad Prism version 8.1 (GraphPad Software, San Diego, California USA, https://www.graphpad.com). The following statistical tests were conducted on all peptides that showed consistent phosphorylation among technical replicates (Chi-squared test, χ^2^ > 0.05). The log_2_-transformed serum *M. hyopneumoniae*-specific IgG titer data and the VSN-transformed kinome data was determined to be normally distributed (Kolmogorov–Smirnov test, p > 0.1). The birth weight of piglets is assumed to follow a normal distribution in nature. The sample size under consideration (n = 117) is large, samples were measured independently, and the mean (1.48 kg) is approximately the median (1.5 kg). Plasma cytokine concentrations were not determined to be normally distributed (Kolmogorov–Smirnov test, p < 0.1). A two-tailed unpaired Student’s t-test was conducted to analyze differences in serum *M. hyopneumoniae*-specific IgG titer, birth weight, weaning weight, or end weight between HR and LR cohorts. A two-tailed Welch’s T-test for Unequal Variances was conducted to determine differences of means between HR and LR phosphorylation intensities as the variances of all peptides did not show homogeneity (F-test of equality of variances, p < 0.05). A two-tailed Mann–Whitney U-test was conducted to analyze differences in plasma cytokine concentrations between HR and LR cohorts. A Pearson linear regression was conducted for the correlation analysis of serum *M. hyopneumoniae*-specific IgG titer and birth weight. A Spearman Rank Correlation was conducted for the correlation analysis of serum *M. hyopneumoniae*-specific IgG titer and plasma IFNγ concentrations, and birth weight and plasma IFNγ concentrations. P-values were considered statistically significant at p < 0.05.

## References

[1] Thornton PK (2010). Livestock production: recent trends, future prospects. Philos. Trans. R. Soc. B Biol. Sci..

[2] Laxminarayan R (2013). Antibiotic resistance—The need for global solutions. Lancet Infect. Dis..

[3] Heininger U (2012). The concept of vaccination failure. Vaccine.

[4] Wang I-M, Bett AJ, Cristescu R, Loboda A, ter Meulen J (2012). Transcriptional profiling of vaccine-induced immune responses in humans and non-human primates. Microb. Biotechnol..

[5] Nakaya HI (2011). Systems biology of vaccination for seasonal influenza in humans. Nat. Immunol..

[6] Rechtien A (2017). Systems vaccinology identifies an early innate immune signature as a correlate of antibody responses to the ebola vaccine rVSV-ZEBOV. Cell Rep..

[7] Bartholomeus E (2018). Transcriptome profiling in blood before and after hepatitis B vaccination shows significant differences in gene expression between responders and non-responders. Vaccine.

[8] Querec TD (2009). Systems biology approach predicts immunogenicity of the yellow fever vaccine in humans. Nat. Immunol..

[9] Tsang JS (2014). Global analyses of human immune variation reveal baseline predictors of postvaccination responses. Cell.

[10] Wilkie B, Mallard B (1999). Selection for high immune response: an alternative approach to animal health maintenance?. Vet. Immunol. Immunopathol..

[11] Newport MJ (2004). Genetic regulation of immune responses to vaccines in early life. Genes Immun..

[12] Posteraro B (2014). The link between genetic variation and variability in vaccine responses: Systematic review and meta-analyses. Vaccine.

[13] Ovsyannikova IG (2011). The role of polymorphisms in Toll-like receptors and their associated intracellular signaling genes in measles vaccine immunity. Hum. Genet..

[14] Dhiman N (2007). Variations in measles vaccine-specific humoral immunity by polymorphisms in SLAM and CD46 measles virus receptors. J. Allergy Clin. Immunol..

[15] Wang C (2004). HLA and cytokine gene polymorphisms are independently associated with responses to hepatitis B vaccination. Hepatology.

[16] Poland GA, Ovsyannikova IG, Jacobson RM (2008). Immunogenetics of seasonal influenza vaccine response. Vaccine.

[17] Frasca D, Blomberg BB (2016). Inflammaging decreases adaptive and innate immune responses in mice and humans. Biogerontology.

[18] Moore SE (2004). Birth weight predicts response to vaccination in adults born in an urban slum in Lahore. Pakistan. Am. J. Clin. Nutr..

[19] Park H-L (2014). Obesity-induced chronic inflammation is associated with the reduced efficacy of influenza vaccine. Hum. Vaccin. Immunother..

[20] Sheridan PA (2012). Obesity is associated with impaired immune response to influenza vaccination in humans. Int. J. Obes..

[21] Burns VE, Carroll D, Ring C, Drayson M (2003). Antibody response to vaccination and psychosocial stress in humans: Relationships and mechanisms. Vaccine.

[22] Eibl N (2002). Impaired Primary immune response in type-1 diabetes: results from a controlled vaccination study. Clin. Immunol..

[23] Crowe SR (2011). Influenza vaccination responses in human systemic lupus erythematosus: Impact of clinical and demographic features. Arthritis Rheum..

[24] Frasca D, Diaz A, Romero M, Landin AM, Blomberg BB (2015). Cytomegalovirus (CMV) seropositivity decreases B cell responses to the influenza vaccine. Vaccine.

[25] Gaucher D (2008). Yellow fever vaccine induces integrated multilineage and polyfunctional immune responses. J. Exp. Med..

[26] Islam MA (2016). Deciphering transcriptome profiles of peripheral blood mononuclear cells in response to PRRSV vaccination in pigs. BMC Genom..

[27] Arsenault RJ (2013). Altered toll-like receptor 9 signaling in *Mycobacterium avium* subsp. paratuberculosis-infected bovine monocytes reveals potential therapeutic targets. Infect. Immun..

[28] Robertson AJ (2014). Identification of developmentally-specific kinotypes and mechanisms of Varroa mite resistance through whole-organism, kinome analysis of honeybee. Front. Genet..

[29] Régnier M (2017). Identification of signaling pathways targeted by the food contaminant FB1: Transcriptome and kinome analysis of samples from pig liver and intestine. Mol. Nutr. Food Res..

[30] Määttänen P (2013). Divergent immune responses to *Mycobacterium avium* subsp. paratuberculosis infection correlate with kinome responses at the site of intestinal infection. Infect. Immun..

[31] Arsenault RJ (2012). *Mycobacterium avium* subsp. paratuberculosis inhibits gamma interferon-induced signaling in bovine monocytes: Insights into the cellular mechanisms of Johne’s disease. Infect. Immun..

[32] Munyaka PM (2019). Characterization of whole blood transcriptome and early-life fecal microbiota in high and low responder pigs before, and after vaccination for *Mycoplasma hyopneumoniae*. Vaccine.

[33] Milligan BN, Dewey CE, de Grau AF (2002). Neonatal-piglet weight variation and its relation to pre-weaning mortality and weight gain on commercial farms. Prev. Vet. Med..

[34] Zimmermann P, Curtis N (2019). Factors that influence the immune response to vaccination. Clin. Microbiol. Rev..

[35] Lambert ND, Ovsyannikova IG, Pankratz VS, Jacobson RM, Poland GA (2012). Understanding the immune response to seasonal influenza vaccination in older adults: A systems biology approach. Expert Rev. Vaccines.

[36] Panda A (2010). Age-associated decrease in TLR function in primary human dendritic cells predicts influenza vaccine response. J. Immunol..

[37] Thompson-Crispi KA (2014). A genome-wide association study of immune response traits in Canadian Holstein cattle. BMC Genom..

[38] Muyanja E (2014). Immune activation alters cellular and humoral responses to yellow fever 17D vaccine. J. Clin. Invest..

[39] Fourati S (2016). Pre-vaccination inflammation and B-cell signalling predict age-related hyporesponse to hepatitis B vaccination. Nat. Commun..

[40] Frasca D (2012). A molecular mechanism for TNF-α-mediated downregulation of B cell responses. J. Immunol..

[41] Frasca D, Diaz A, Romero M, Landin AM, Blomberg BB (2014). High TNF-α levels in resting B cells negatively correlate with their response. Exp. Gerontol..

[42] Schoenborn JR, Wilson CB (2007). Regulation of interferon-γ during innate and adaptive immune responses. Adv. Immunol..

[43] Parronchi P (1997). Type 1 T-helper cell predominance and interleukin-12 expression in the gut of patients with Crohn’s disease. Am. J. Pathol..

[44] Kumar P (2012). Interferon γ and glycemic status in diabetes associated with chronic pancreatitis. Pancreatology.

[45] Harigai M (2008). Excessive production of IFN-γ in patients with systemic lupus erythematosus and its contribution to induction of b lymphocyte stimulator/B cell-activating factor/TNF ligand superfamily-13B. J. Immunol..

[46] Pellanda LC (2009). Low birth weight and markers of inflammation and endothelial activation in adulthood: the ARIC study. Int. J. Cardiol..

[47] Hagan T (2019). Antibiotics-driven gut microbiome perturbation alters immunity to vaccines in humans. Cell.

[48] Diaz A (2017). Metformin improves in vivo and in vitro B cell function in individuals with obesity and Type-2 diabetes. Vaccine.

[49] Alter G, Sekaly RP (2015). Beyond adjuvants: Antagonizing inflammation to enhance vaccine immunity. Vaccine.

[50] Quiniou N, Dagorn J, Gaudré D (2002). Variation of piglets’ birth weight and consequences on subsequent performance. Livest. Prod. Sci..

[51] Milligan BN, Fraser D, Kramer DL (2002). Within-litter birth weight variation in the domestic pig and its relation to pre-weaning survival, weight gain, and variation in weaning weights. Livest. Prod. Sci..

[52] Han K (2012). Revaccination of non- and low-responders after a standard three dose hepatitis B vaccine schedule. Hum. Vaccin. Immunother..

[53] McDade TW, Beck MA, Kuzawa C, Adair LS (2001). Prenatal undernutrition, postnatal environments, and antibody response to vaccination in adolescence. Am. J. Clin. Nutr..

[54] McDade TW, Adair L, Feranil AB, Kuzawa C (2011). Positive antibody response to vaccination in adolescence predicts lower C-reactive protein concentration in young adulthood in the Philippines. Am. J. Hum. Biol..

[55] Arsenault R, Griebel P, Napper S (2011). Peptide arrays for kinome analysis: New opportunities and remaining challenges. Proteomics.

[56] Krijthe, J. H. *Rtsne: T-distributed stochastic neighbor embedding using barnes-hut implementation*. R package version 0.15. https://github.com/jkrijthe/Rtsne (2015).

[57] Wickham H (2016). ggplot2: Elegant Graphics for Data Analysis.

[58] Li Y (2012). A systematic approach for analysis of peptide array kinome data. Sci. Signal..

[59] Breuer K (2013). InnateDB: Systems biology of innate immunity and beyond–recent updates and continuing curation. Nucleic Acids Res..

